# Supplementary figures and images for: Influence of Butyrate on Impaired Gene Expression in Colon from Patients with High Blood Pressure
Source: Int J Mol Sci. 2023 Jan 31;24(3):2650. doi: 10.3390/ijms24032650 (PMC9917256; doi:10.3390/ijms24032650)

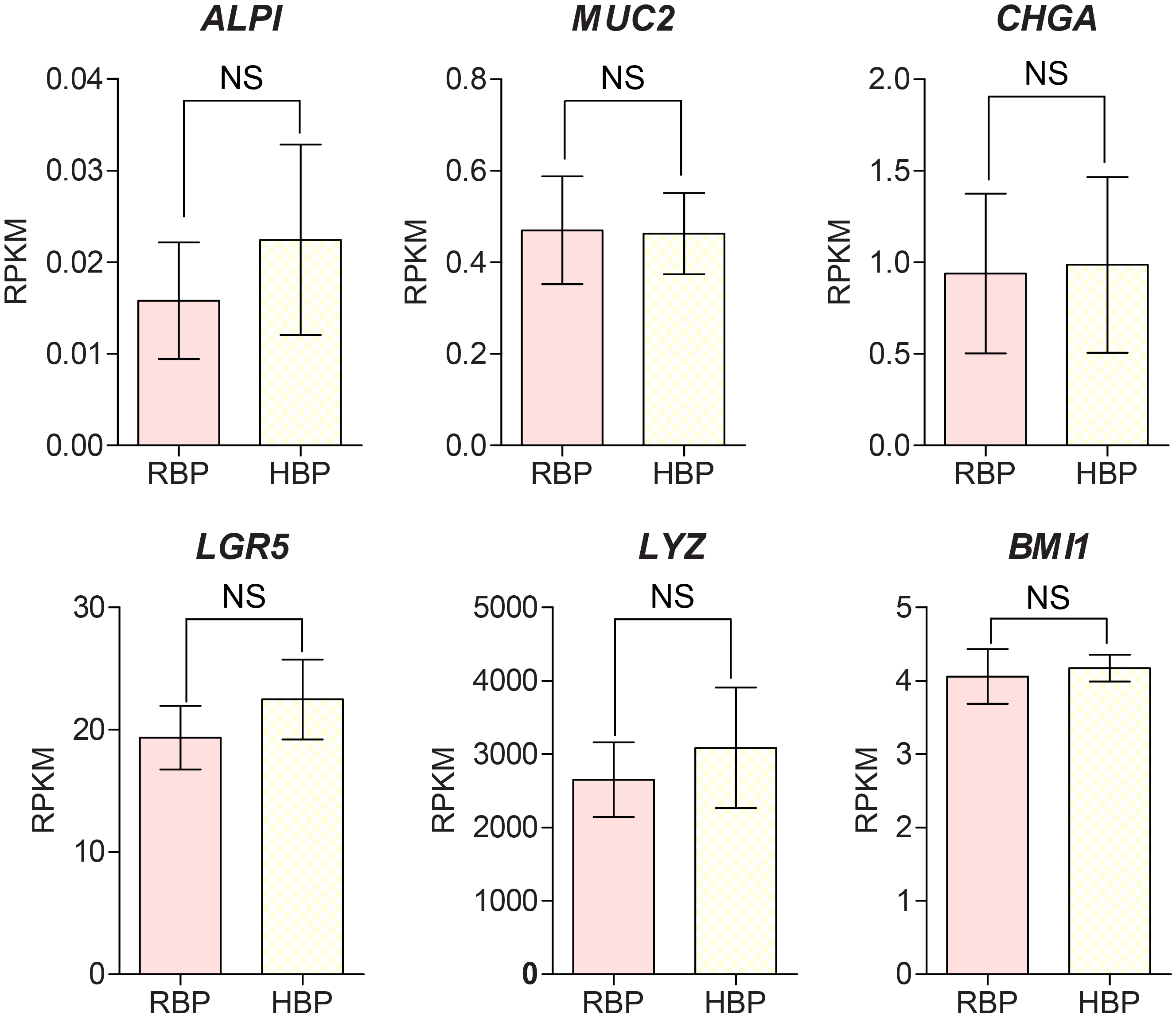

Supplement: Supplementary file 1 [file ijms-24-02650-s001.zip › ijms-2137893-supplementary.png]
